# Molecular Mechanism of Selective Binding of NMS-P118 to PARP-1 and PARP-2: A Computational Perspective

**DOI:** 10.3389/fmolb.2020.00050

**Published:** 2020-04-15

**Authors:** Ran Wang, Yalong Cong, Mengxin Li, Jinxiao Bao, Yifei Qi, John Z. H. Zhang

**Affiliations:** ^1^Shanghai Key Laboratory of Green Chemistry and Chemical Process, Shanghai Engineering Research Center of Molecular Therapeutics and New Drug Development, School of Chemistry and Molecular Engineering, East China Normal University at Shanghai, Shanghai, China; ^2^NYU-ECNU Center for Computational Chemistry at NYU Shanghai, Shanghai, China; ^3^Department of Chemistry, New York University, New York, NY, United States; ^4^Collaborative Innovation Center of Extreme Optics, Shanxi University, Taiyuan, China

**Keywords:** PARP, NMS-P118, selectivity, interaction entropy, binding free energy, alanine scanning

## Abstract

The nuclear protein poly (ADP-ribose) polymerase-1 (PARP-1) inhibitors have been proven effective to potentiate both chemotherapeutic agents and radiotherapy. However, a major problem of most current PARP inhibitors is their lack of selectivity for PARP-1 and its closest isoform PARP-2. NMS-P118 is a highly selective PARP inhibitor that binds PARP-1 stronger than PARP-2 and has many advantages such as excellent pharmacokinetic profiles. In this study, molecular dynamics (MD) simulations of NMS-P118 in complex with PARP-1 and PARP-2 were performed to understand the molecular mechanism of its selectivity. Alanine scanning together with free energy calculation using MM/GBSA and interaction entropy reveal key residues that are responsible for the selectivity. Although the conformation of the binding pockets and NMS-P118 are very similar in PARP-1 and PARP-2, most of the hot-spot residues in PARP-1 have stronger binding free energy than the corresponding residues in PARP-2. Detailed analysis of the binding energy shows that the 4′4-difluorocyclohexyl ring on NMS-P118 form favorable hydrophobic interaction with Y889 in PARP-1. In addition, the H862 residue in PARP-1 has stronger binding free energy than H428 in PARP-2, which is due to shorter distance and stronger hydrogen bonds. Moreover, the negatively charged E763 residue in PARP-1 forms stronger electrostatic interaction energy with the positively charged NMS-P118 than the Q332 residue in PARP-2. These results rationalize the selectivity of NMS-P118 and may be useful for designing novel selective PARP inhibitors.

## Introduction

DNA damage occurs constantly in organisms and may lead to disease if not repaired in time (Hosoya and Miyagawa, 2009). Therefore, cells have evolved sophisticated DNA damage detection and repair systems to maintain normal physiological functions. Blocking the DNA repair pathway has become important in many anti-cancer chemotherapeutic drugs (Staibano et al., 2005) that aim to kill tumor cells by damaging DNA (Jones and Plummer, 2008; Drew and Plummer, 2009).

Poly (ADP-ribose) polymerase (PARP) is an enzyme that locates in the nucleus and is involved in the synthesis of poly ADP-ribose, a multimer of ADP-ribose linked by ribosylation-ribose bonds. Since the discovery of the first poly ADP ribose polymer (PAR) (Sabau et al., 1999; Qiu et al., 2014), at least 17 members of the PARP family that share a homologous catalytic region have been identified (Peralta-Leal et al., 2009). The PARP family has gained much attention because of their involvement in genomic stability, DNA damage response, apoptosis by DNA damage, cell division, transcriptional regulation, and chromatin remodeling (Ame et al., 2004; Schreiber et al., 2006; Hakmé et al., 2008; Anwar et al., 2015). PARP-1 is the first discovered and most thoroughly studied family member with core repair function during base excision repair (BER) (Bennett and Xie, 1988). When DNA single-strand break occurs in the cell, PARP-1 actively participates in the repair process with BER (Lord and Ashworth, 2017; Berger et al., 2018). Inhibition of PARP-1 impairs the DNA damage repair and causes apoptosis of cancer cells, and therefore reduces the intensity of chemotherapy and eventually the damage to human body (Davar et al., 2012; Lupo and Trusolino, 2014). So far, many potent PARP-1 inhibitors such as olaparib (Rottenberg et al., 2008), rucaparib (Tikhe et al., 2004; Thomas et al., 2007), tatzoparib (Timonen et al., 2011; Shen et al., 2013), and veliparib (Donawho et al., 2007) have been discovered. However, an important problem of these inhibitors is the lack of selectivity to PARP-1 and PARP-2 (Papeo et al., 2015).

PARP-2 is the closest homolog of PARP-1 and shares 84% identity and 90% similarity within the PARP signature motif (Krishnakumar and Kraus, 2010), it only contribution 5–10% to the total DNA damage induced PARP activity (Yélamos et al., 2008). Moreover inhibition of PARP-2 would affect important cellular functions such as genome surveillance, spermatogenesis, adipogenesis, and T cell development (Yélamos et al., 2008; Papeo et al., 2015). However, all PARP-1 inhibitors discovered so far also inhibit PARP-2. Therefore, it is important to develop efficient selective PARP-1 inhibitors that do not bind PARP-2.

**Figure 1:**
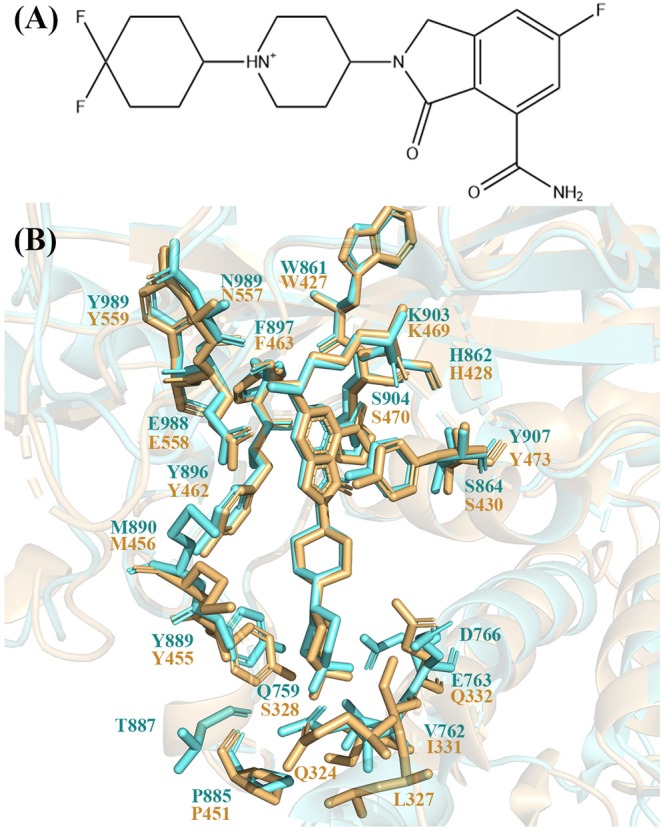
**(A)** 2D skeleton structure of the compound NMS-P118. The plus sign indicates the positive charge on the N atom in the 1,4-dimethylpiperidin-1-ium group. **(B)** The binding pocket of NMS-P118 in PARP-1 and PARP-2. The PARP-1 is colored with aquamarine and PARP-2 is colored with light orange.

Recently, a potent, orally available, and highly selective PARP-1 inhibitor named NMS-P118 was discovered and in preclinical studies for cancer therapy (Figure 1A). It has a selective ratio (PARP-2 Kd/PARP-1 Kd) of 154 and mitigates toxicities arising from cross-inhibition of PARP-2 with excellent pharmacokinetic profiles and high oral availability in mouse and rat (Papeo et al., 2015). Quantitative understanding of the different binding modes of NMS-P118 to PARP-1 and PARP-2 is important to design novel selective PARP-1 inhibitors. However, the crystal structure of PARP-1 and PARP-2 show very similar binding pose with a pocket RMSD of 0.80 Å (Figure 1B). Therefore, it is difficult to rationalize the selectivity based on the static crystal structures alone.

Computational simulation and accurate calculation of protein-ligand binding free energy is a powerful approach in understanding ligand binding mechanisms from a dynamic perspective (Sun et al., 2013). Although some methods such as free energy perturbation (FEP) (Bash et al., 1987; Rao et al., 1987; Kollman, 1993; Kita et al., 1994; Jorgensen and Thomas, 2008) and thermodynamic integration (TI) (Beveridge and DiCapua, 1989; Zacharias et al., 1994) are rigorous for binding free energy calculation, they are not routinely used due to high computational demand for large protein-ligand system. On the other hand, the Molecular Mechanics/Generalized Born Surface Area (MM/GBSA) is a widely used method because of its efficiency in the absolute free energy calculation (Massova and Kollman, 1999, 2000; Kollman et al., 2000; Moreira et al., 2007; Sun et al., 2014). It is important to note that in MM/GBSA calculation, the entropy contribution can be obtained by the normal mode approach (Nguyen and Case, 1985; Brooks et al., 1995) which is often neglected because of its high computational cost and rough accuracy (Sun et al., 2018). On this regard, a recent method called interaction entropy (IE) (Duan et al., 2016) for calculating entropy contribution has been developed and successfully used in protein-ligand and protein-protein systems (Cong et al., 2017; Yan et al., 2017; Liu et al., 2018).

In general, only a few important residues play a major role in protein-ligand binding (Burgoyne and Jackson, 2006; Barillari et al., 2008; Cheung et al., 2012; Bauman et al., 2016). Therefore, it is crucial to identify these key residues and explore the binding mechanism of protein-ligand to improve the binding potency and selectivity of ligand. To this end, the alanine scanning (AS) approach has been combined with the MM/GBSA_IE method to obtain binding free energies of specific residues (Yan et al., 2017; Qiu et al., 2018). In this approach, a single-trajectory MD simulation is performed starting from the initial complex structure. Alanine scanning is then carried out on individual residues in the trajectory and the enthalpy and entropic component are calculated with the MM/GBSA and IE method, respectively (Gohlke et al., 2000).

In this study, to gain quantitative understanding of the selectivity of NMS-P118, MD simulations were performed on the complex structures of NMS-P118 with PARP-1 and PARP-2. Binding free energy calculation was carried out and showed that NMS-P118 binds PARP-1 3.04 kcal/mol stronger than PARP-2, which agrees with the experimental affinity. Based on our calculations, the hot-spot residues that are responsible for the different binding affinity were identified, and the detailed molecular interactions were analyzed. These results explain the selectivity of NMS-P118 and may provide guidance for future study and design of novel PARP inhibitors.

## Method

### MD Simulation

The initial structures for MD simulation and alanine scanning were obtained from the Protein Databank (PDB) (Berman et al., 2000). The PDB IDs for PARP-1-NMS-P118 and PARP-2-NMS-P118 were 5A00 and 4ZZY (Papeo et al., 2015). The AMBER ff14SB force field (Maier et al., 2015) and the general AMBER force field (GAFF) (Wang et al., 2004) were used to parameterize the system. The complexes were solvated in truncated octahedron TIP3P (Jorgensen et al., 1983) boxes with a 10.0 Å buffer distance. Chloride and sodium ions were added as counterbalance ions to neutralize the system. To eliminate the bad contacts between solute and solvent water molecule, we used the conjugate gradient minimization followed by the steepest descent method to minimize the energy. During minimization, the solvent water molecules were optimized first by restraining the coordinates of other molecules with a force constant of 500 kcal/(mol *Å^2^). The restrains were then removed in a subsequent minimization step to optimized the whole system.

The minimized system was slowly heated to 300 K within 300 ps with all solute atoms restrained with a force constant of 10 kcal/(mol *Å^2^). After equilibration at 300 K, a production simulation was carried out in NPT ensemble for 100 ns, where the Langevin dynamics was used to maintain the temperature and Berendsen barostat was used to control the pressure at 1.0 atm. All bonds involving hydrogen atoms were constrained by the SHAKE (Ryckaert et al., 1977), and the cutoff distance for the non-bonded interactions was 10.0 Å. The simulation time step was 2 fs and the trajectory were recorded every 1 ps. Each complex structure was simulated with three 100 ns replicates. Hydrogen bond was identified with a distance cutoff of 3.5 Å and angle cutoff of 120°.

### Computational Alanine Scanning

**Figure 2:**
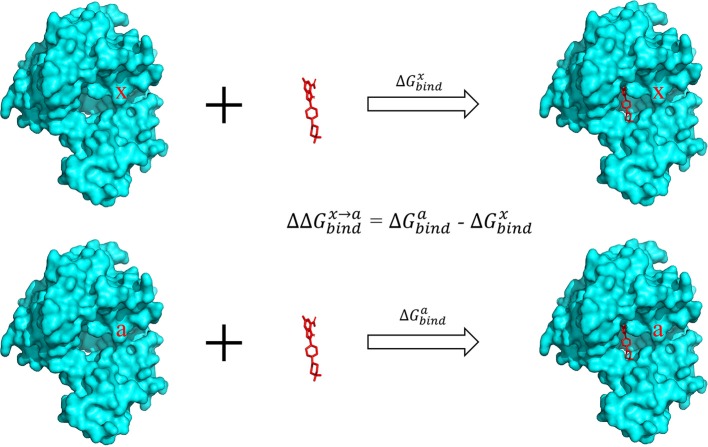
Calculation of binding free energy with the alanine scanning approach. The contribution of a specific residue x in the pocket was calculated by mutating this residue to alanine and calculating the difference of the binding energy in the two complexes.

The alanine scanning method was used to calculate the binding free energy. In this approach, a residue was mutated to alanine and the difference of binding free energy before and after the mutation was calculated (Figure 2). In generally, the mutated alanine has negligible contribution to the binding free energy. Therefore, the difference of binding free energy before and after the mutation is equivalent to the contribution of the mutated residue to the total binding free energy.

The difference of binding free energy between wild type (WT) and alanine-mutated complex structure is defined by the following formula:

(1) $$\begin{array}{l} \begin{array}{c} \Delta \Delta G_{bind}^{x\to a}=\Delta G_{bind}^{a}-\Delta G_{bind}^{x} \end{array} \end{array}$$

where

(2) $$\begin{array}{l} \Delta G_{bind}^{x}=\Delta G\left(P\left(x\right)-L\right)\\ \;=\Delta G_{gas}\left(P\left(x\right)-L\right)+\Delta G_{sol}\left(P\left(x\right)-L\right) \end{array}$$

and similar for $\Delta G_{bind}^{a}$

(3) $$\begin{array}{l} \Delta G_{bind}^{a}=\Delta G\left(P\left(a\right)-L\right)\\ \;=\Delta G_{gas}\left(P\left(a\right)-L\right)+\Delta G_{sol}\left(P\left(a\right)-L\right) \end{array}$$

In the above equations, “P” and “L” represent the protein and ligand, respectively. The superscript “x” represents a residue on the surface of protein, and superscript “a” represents the mutation of residue x into alanine. $\Delta G_{bind}^{x}$ is the binding free energy of wild type complex while $\Delta G_{bind}^{a}$ is the binding free energy of the mutant. *P*(*x*) and *P*(*a*) represent the wildtype residue x and the mutated alanine in the protein. Δ*G*_*gas*_(*P*(*x*) − *L*) and Δ*G*_*sol*_(*P*(*x*) − *L*) represent the gas-phase component and the solvation free energy component of total binding free energy of the wild-type, similarly to the mutant.

Hence, formula (1) can be written as

(4) $$\begin{array}{ll} \Delta \Delta G_{bind}^{x\to a}=\Delta G_{gas}\left(P\left(a\right)-L\right)+\Delta G_{sol}\left(P\left(a\right)-L\right)-\Delta G_{gas}\left(P\left(x\right)-L\right)\\ - & \Delta G_{sol}\left(P\left(x\right)-L\right)\\ =\left[\Delta G_{gas}\left(P\left(a\right)-L\right)-\Delta G_{gas}\left(P\left(x\right)-L\right)\right]\\ + & \left[\Delta G_{sol}\left(P\left(a\right)-L\right)-\Delta G_{sol}\left(P\left(x\right)-L\right)\right]\\ =\Delta \Delta G_{gas}^{x\to a}+\Delta \Delta G_{sol}^{x\to a} \end{array}$$

Here, $\Delta \Delta G_{bind}^{x\to a}$ is the difference of binding free energy before and after mutation. The relative binding free energy can be divided into a gas-phase term $\Delta \Delta G_{gas}^{x\to a}$and a solvation term $\Delta \Delta G_{sol}^{x\to a}$.

The gas phase $\Delta \Delta G_{gas}^{x\to a}$ can be obtained in the following way:

(5) $$\begin{array}{l} \begin{array}{c} \Delta \Delta G_{gas}^{x\to a}\approx \Delta G_{gas}^{a}\left(a-L\right)-\Delta G_{gas}^{x}\left(x-L\right)\; \end{array} \end{array}$$

Here, $\Delta G_{gas}^{a}\left(a-L\right)$ and $\Delta G_{gas}^{x}\left(x-L\right)$ indicates the gas-phase binding free energy of the mutated alanine and the wildtype residue to the ligand, respectively. During alanine scanning, other residues remained unchanged, so the difference between $\Delta G_{gas}^{a}\left(a-L\right)$ and $\Delta G_{gas}^{x}\left(x-L\right)$ is the contribution of x residue to the total binding free energy.

The solvation energy was obtained with the following formula

(6) $$\begin{array}{l} \begin{array}{c} \Delta G_{sol}\left(P\left(x\right)-L\right)=G_{sol}\left(P\left(x\right)-L\right)-G_{sol}\left(P\left(x\right)\right)-G_{sol}\left(L\right) \end{array} \end{array}$$

Here *G*_*sol*_(*P*(*x*) − *L*) represents the solvation energy of *P*(*x*) − *L* complex, and similar for *G*_*sol*_(*P*(*a*) − *L*).

Finally, the difference of solvation energy becomes

(7) $$\begin{array}{l} \Delta \Delta G_{sol}^{x\to a}=G_{sol}\left(P\left(a\right)-L\right)-G_{sol}\left(P\left(a\right)\right)\\ \;-G_{sol}\left(L\right)-G_{sol}\left(P\left(x\right)-L\right)+G_{sol}\left(P\left(x\right)\right)+G_{sol}(L)\\ \;=G_{sol}\left(P\left(a\right)-L\right)-G_{sol}\left(P\left(a\right)\right)-G_{sol}\left(P\left(x\right)-L\right)\\ \;+G_{sol}\left(P\left(x\right)\right)\\ \;=G_{sol}\left(P\left(a\right)-L\right)-G_{sol}\left(P\left(x\right)-L\right)-[G_{sol}\left(P\left(a\right)\right)\\ \;-G_{sol}\left(P\left(x\right)\right)] \end{array}$$

Where the *G*_*sol*_(*P*(*x*) − *L*) means the solvation energy of wild type complex P(x)-L, and *G*_*sol*_(*P*(*x*)) means the solvation energy of wild type protein.

### Interaction Entropy Method

The gas-phase component of binding free energy is composed of two parts: molecular mechanics interaction energy and entropic contribution. In this study, the entropy was obtained by the IE method which is more efficient than the conventional normal mode method (Duan et al., 2016; Yan et al., 2017; Liu et al., 2018). The gas-phase component of the binding free energy is derived as follows:

(8) $$\begin{array}{l} \Delta G_{gas}=-kT\ln\frac{\int dq_{w}dq_{p}dq_{l}e^{-\beta \left(E^{p}+E^{l}+E_{int}^{pl}+E^{w}+E_{int}^{pw}+E_{int}^{lw}\right)}}{\int dq_{w}dq_{p}dq_{l}e^{-\beta \left(E^{p}+E^{l}+E^{w}+E_{int}^{pw}+E_{int}^{lw}\right)}}\\ =-kT\ln\left[\frac{1}{\langle e^{\beta E_{int}^{pl}}\rangle }\right]=kT\ln\left[\langle e^{\beta E_{int}^{pl}}\rangle \right]\\ =\;kT\ln\left[e^{\beta \langle E_{int}^{pl}\rangle }\langle e^{\beta \left(E_{int}^{pl}-\langle E_{int}^{pl}\rangle \right)}\rangle \right]\;\\ =\langle E_{int}^{pl}\rangle +kT\ln\langle e^{\beta \Delta E_{int}^{pl}}\rangle \\ =\langle E_{int}^{pl}\rangle -T\Delta S \end{array}$$

*E*^*p*^, *E*^*l*^, and *E*^*w*^ repsesent internal energies of protein, ligand, and waters, respectively. $E_{int}^{pl}$, $E_{int}^{pw}$, and $E_{int}^{lw}$ are interaction energies of protein-ligand, protein-water, and ligand-water, respectively. $\Delta E_{int}^{pl}=E_{int}^{pl}-\langle E_{int}^{pl}\rangle$ represents the fluctuation of the energy and therefore:

(9) $$\begin{array}{l} \begin{array}{c} -T\Delta S=kT\ln\langle e^{\beta \Delta E_{int}^{pl}}\rangle \end{array} \end{array}$$

Thus, $\Delta G_{gas}^{x}\;$can be calculated by the following formula:

(10) $$\begin{array}{l} \Delta G_{gas}^{x}=\langle E_{int}^{x}\rangle -T\Delta S_{int}^{x}\\ \;=\langle E_{int}^{x}\rangle +kT\ln\langle e^{\beta \Delta E_{int}^{x}}\rangle \end{array}$$

Based on the IE method:

(11) $$\begin{array}{l} \begin{array}{c} -T\Delta S_{int}^{x}=kT\ln\langle e^{\beta \Delta E_{int}^{x}}\rangle \end{array} \end{array}$$

and similar for $\Delta G_{gas}^{a}$

(12) $$\begin{array}{l} \Delta G_{gas}^{a}=\langle E_{int}^{a}\rangle -T\Delta S_{int}^{a}\;\\ \;=\langle E_{int}^{a}\rangle +kT\ln\langle e^{\beta \Delta E_{int}^{a}}\rangle \; \end{array}$$

Among the formulas above, $\Delta E_{int}^{x}=E_{int}^{x}-\langle E_{int}^{x}\rangle$ represents the fluctuation of the energy. The average of *E*_*int*_ was calculated on the MD trajectory:

(13) $$\begin{array}{l} \langle E_{int}\rangle \;=\;\frac{1}{T}\int_{0}^{T}E_{int}\left(t\right)dt=\frac{1}{N}\overset{N}{\underset{i=1}{\sum }}E_{int}\left(t_{i}\right) \end{array}$$

therefore

(14) $$\begin{array}{l} \begin{array}{c} \langle e^{\beta \Delta E_{int}^{x}}\rangle =\frac{1}{N}\sum_{i=1}^{N}e^{\beta \Delta E_{int}^{x}\left(t_{i}\right)\;} \end{array} \end{array}$$

and similar for the mutated trajectory:

(15) $$\begin{array}{l} \begin{array}{c} \langle e^{\beta \Delta E_{int}^{a}}\rangle \;=\;\frac{1}{N}\sum_{i=1}^{N}e^{\beta \Delta E_{int}^{a}\left(t_{i}\right)} \end{array} \end{array}$$

Finally, the difference of gas phase binding free energy is

(16) $$\begin{array}{l} \begin{array}{c} \Delta \Delta G_{gas}^{x\to a}=\langle E_{int}^{a}\rangle -\langle E_{int}^{x}\rangle +kT\left[\ln\langle e^{\beta \Delta E_{int}^{a}}\rangle -\ln\langle e^{\beta \Delta E_{int}^{x}}\rangle \right] \end{array} \end{array}$$

### MM/GBSA Method

For the solvation energy,

(17) $$\begin{array}{ll} \Delta \Delta G_{sol}^{x\to a}=G_{sol}\left(P\left(a\right)-L\right)-G_{sol}\left(P\left(x\right)-L\right)\\ \;- & \left[G_{sol}\left(P\left(a\right)\right){\;-\;G}_{sol}\left(P\left(x\right)\right)\right] \end{array}$$

The solvation energy was calculated with the GBSA method

(18) $$\begin{array}{l} \begin{array}{c} \Delta G_{sol}=\Delta G_{gb}+\Delta G_{np} \end{array} \end{array}$$

Here, Δ*G*_*gb*_, Δ*G*_*np*_ represent the generalize-born and non-polar solvation free energy, respectively.

Δ*G*_*gb*_ is obtained by OBC GBSA model with igb = 2 (Ryckaert et al., 1977; Onufriev et al., 2000, 2004), with the dielectric constants of 1, 3, and 10 for non-polar, polar and charged residues, respectively (Qiu et al., 2018; Li et al., 2020). Δ*G*_*np*_ is obtained by empirical solvent-accessible surface area formula:

(19) $$\begin{array}{l} \begin{array}{c} \Delta G_{np}=\gamma SASA+\beta \; \end{array} \end{array}$$

In this study, the constants γ and β adopted the values of 0.00542 *kcal*/(mol^*^Å^2^) and 0.92 kcal/mol, respectively.

The total binding free energy was calculated by summation of the contribution of residues within 5 Å of the ligand in the crystal structure:

(20) $$\begin{array}{l} \begin{array}{c} \Delta G_{bind}=-\sum_{x}\Delta \Delta G_{bind}^{x\to a} \end{array} \end{array}$$

### Binding Free Energy Difference ($\Delta \Delta G_{bind}^{x\to a}$) in Alanine Scanning

Five 5-ns windows that have a stable complex structure were selected from each of the 100-ns trajectories to calculate binding free energy. Therefore, the reported energy values were averaged over 15 windows. The mutant trajectories were obtained by removing sidechain atoms that are beyond the *C*_β_ atom in the wild-type trajectories. The solvation free energy was calculated using the GB model with *igb* = 2. There are 100 equally distributed frames in every 5-ns window were extracted for calculation of enthalpy. Entropy was obtained by Equation (11) using all 5,000 frames for every 5-ns window. To eliminate noises and ensure convergence, energy values that are within three standard deviation of the average value were used to calculate IE (Qiu et al., 2018).

## Results and Discussion

### Stability of the Complex Systems

**Figure 3:**
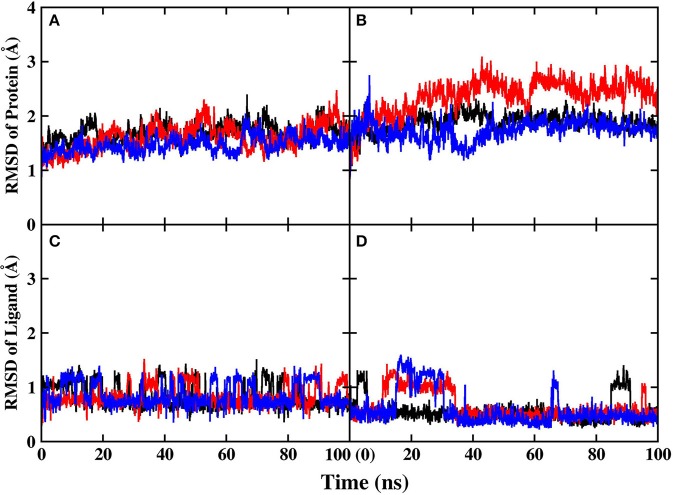
RMSD of protein and ligand in MD simulations. Different colors represent different replicates. **(A)** Protein backbone in PARP-1 **(B)** protein backbone in PARP-2 **(C)** ligand in PARP-1 **(D)** ligand in PARP-2.

Before performing energy calculations and alanine scanning, the root-mean-square-deviation (RMSD) of the protein backbone atoms and ligand with respect to the initial crystal structure was calculated to exam whether the systems are stable in the MD simulations (Figure 3).

**Figure 4:**
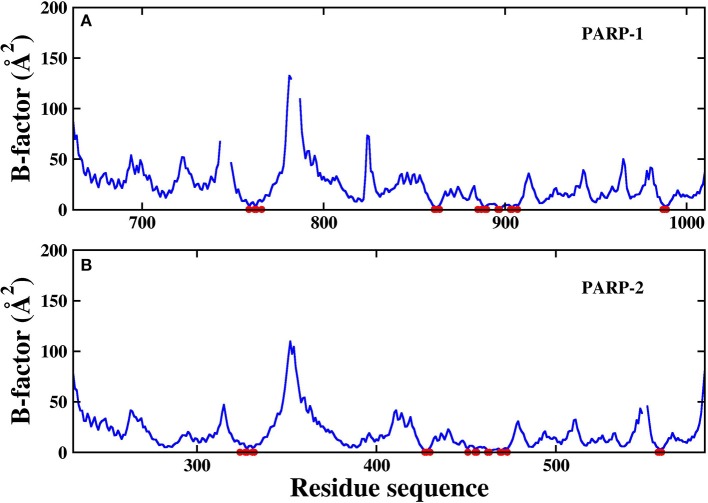
The calculated B-factor of protein *C*_α_ atoms in the simulations. The residues in the pocket are marked with red circle. **(A)** PARP-1 **(B)** PARP-2.

The RMSD of the protein backbone in PARP-1 is around 1.5 Å and the RMSD in PARP-2 is around 2.0 Å. The RMSD of ligand in PARP-1 and PARP-2 is <1 Å in most of the time, although there are some fluctuations due to small conformational change of the ligand. Besides, the isotropic temperature factor (B-factor) was calculated to reveal the mobility of each residue around its mean position in the simulations (Figure 4). The B-factor values of the residues in the pocket are relatively low, which shows that they are very stable throughout the simulation. These results suggest that the complex structures are overall stable in the simulations.

**Figure 5:**
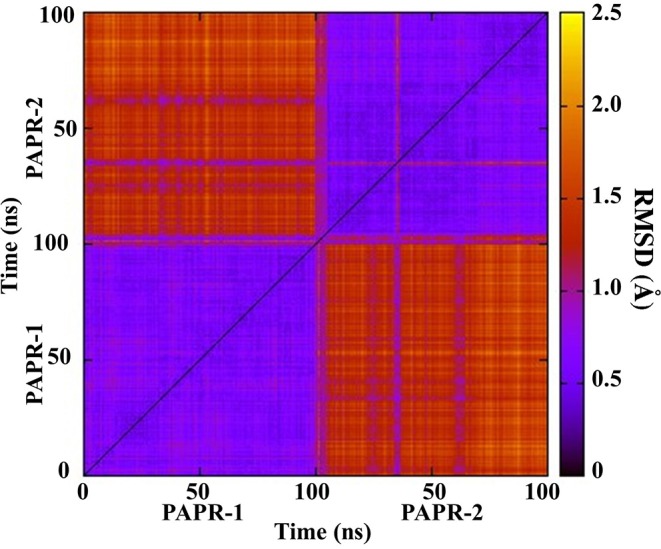
2D-RMSD of PARP-1 and PARP-2 during simulations.

To exam the difference between the binding pockets of PARP-1 and PARP-2 during simulation, the 2D-RMSD of backbone atoms in the binding pockets is calculated (Figure 5). It is clear that the similarity of the PARP-1 and PARP-2 pockets are decreased during simulation, and suggests that it is important to study the binding interaction from a dynamic perspective instead of a static structure.

### Comparisons Between Calculated and Experimental Binding Free Energy

Five stable 5-ns segments from each trajectory are selected for the calculation of binding free energy. The computational alanine scanning was carried out using the MM/GBSA_IE method on the residues that are within 5 Å of the ligand in the two complexes. The total binding free energy is obtained by adding up binding free energy of each pocket residue (Table 1). The calculated binding free energy of PAPP-1 is 3.04 kcal/mol stronger than that of PARP-2, which is consistent with the experimental value of 3.00 kcal/mol.

**Table 1:** Overall binding free energy of NMS-P118 with PARP-1 and PARP-2 using the MM/GBSA_IE method.

	**PARP-1**			**PARP-2**			**Difference**
	**ΔH**	**−*T*Δ*S***	**ΔG**	**ΔH**	**−*T*Δ*S***	**ΔG**	
Mean	−24.31	5.11	−19.20	−22.29	6.13	−16.16	3.04
SD	0.43	0.53	0.48	0.43	0.83	0.85	
Δ*G*_exp_			−10.90			−7.90	3.00

*All energy values are given in kcal/mol. SD represents the Standard Deviation*.

### Residue-Specific Binding Free Energies

To explore the reasons for the stronger binding ability of PARP-1, the contribution of binding free energy of each pocket residue was analyzed to study the differences between PARP-1 and PARP-2 (Table 2). The residue-specific binding free energy were obtained by MM/GBSA_IE method and averaged on the 15 MD segments for each system. Residues that contribute more than 1 kcal/mol are classified as hot-spot residues.

**Table 2:** Computational alanine scanning for PARP-1 and PARP-2 with the MM/GBSA_IE method.

**PARP-1**							
**Mutation**	**ΔΔ*E*_*vdw*_**	**ΔΔ*E*_*ele*_**	**ΔΔ*G*_*gb*_**	**ΔΔ*G*_*np*_**	**ΔΔ*H***	**−*T*ΔΔ*S***	**ΔΔ*G***
Y907A	6.41 ± 0.15	−0.09 ± 0.19	−0.25 ± 0.24	0.27 ± 0.04	6.33 ± 0.15	−0.73 ± 0.13	5.60 ± 0.26
Y889A	4.05 ± 0.14	0.09 ± 0.30	−0.04 ± 0.26	0.24 ± 0.02	4.35 ± 0.18	−0.66 ± 0.23	3.69 ± 0.24
Y896A	3.33 ± 0.09	0.90 ± 0.05	−0.86 ± 0.04	0.13 ± 0.01	3.51 ± 0.09	−0.41 ± 0.05	3.10 ± 0.13
H862A	1.53 ± 0.16	−0.44 ± 0.12	0.67 ± 0.12	0.06 ± 0.00	1.81 ± 0.16	−0.17 ± 0.04	1.64 ± 0.19
E988A	1.04 ± 0.13	4.94 ± 0.11	−4.43 ± 0.10	0.09 ± 0.02	1.65 ± 0.14	−0.28 ± 0.11	1.37 ± 0.25
E763A	1.20 ± 0.21	4.90 ± 0.28	−4.43 ± 0.27	0.16 ± 0.04	1.83 ± 0.25	−0.87 ± 0.29	0.96 ± 0.23
K903A	1.38 ± 0.21	−3.59 ± 0.23	3.15 ± 0.20	0.07 ± 0.02	1.00 ± 0.17	−0.13 ± 0.05	0.87 ± 0.16
D766A	0.19 ± 0.08	4.00 ± 0.21	−3.54 ± 0.20	0.01 ± 0.01	0.67 ± 0.10	−0.15 ± 0.08	0.52 ± 0.03
V762A	0.56 ± 0.03	−0.19 ± 0.02	0.19 ± 0.02	0.03 ± 0.01	0.60 ± 0.03	−0.11 ± 0.03	0.49 ± 0.05
Q759A	0.84 ± 0.05	0.35 ± 0.26	−0.41 ± 0.23	0.05 ± 0.02	0.83 ± 0.04	−0.39 ± 0.31	0.44 ± 0.30
F897A	0.23 ± 0.01	0.24 ± 0.02	−0.18 ± 0.03	0.00 ± 0.00	0.30 ± 0.02	−0.02 ± 0.01	0.28 ± 0.02
S904A	−0.55 ± 0.29	1.87 ± 0.29	−0.58 ± 0.14	0.00 ± 0.00	0.74 ± 0.13	−0.46 ± 0.14	0.28 ± 0.21
W861A	0.06 ± 0.00	−0.01 ± 0.02	0.12 ± 0.01	0.00 ± 0.00	0.17 ± 0.01	0.00 ± 0.00	0.17 ± 0.01
T887A	0.16 ± 0.06	0.12 ± 0.15	−0.09 ± 0.13	0.01 ± 0.00	0.20 ± 0.09	−0.03 ± 0.04	0.17 ± 0.10
Y989A	0.07 ± 0.01	−0.02 ± 0.01	0.05 ± 0.01	0.00 ± 0.00	0.10 ± 0.01	0.00 ± 0.00	0.10 ± 0.01
P885A	0.04 ± 0.02	0.09 ± 0.02	−0.08 ± 0.02	0.00 ± 0.00	0.05 ± 0.02	−0.01 ± 0.01	0.04 ± 0.02
N987A	0.01 ± 0.00	0.02 ± 0.02	−0.02 ± 0.02	0.00 ± 0.00	0.01 ± 0.00	0.00 ± 0.00	0.01 ± 0.00
S864A	0.03 ± 0.01	−0.05 ± 0.12	0.03 ± 0.12	0.00 ± 0.00	0.02 ± 0.01	−0.02 ± 0.02	0.00 ± 0.02
**PARP-2**							
**Mutation**	**ΔΔ*E*_*vdw*_**	**ΔΔ*E*_*ele*_**	**ΔΔ*G*_*gb*_**	**ΔΔ*G*_*np*_**	**ΔΔ*H***	**−*T*ΔΔ*S***	**ΔΔ*G***
Y473A	6.34 ± 0.13	0.18 ± 0.10	−0.55 ± 0.11	0.34 ± 0.01	6.31 ± 0.12	−0.91 ± 0.08	5.40 ± 0.16
Y455A	3.35 ± 0.70	0.32 ± 0.25	−0.18 ± 0.24	0.19 ± 0.08	3.67 ± 0.76	−0.53 ± 0.19	3.14 ± 0.58
Y462A	3.40 ± 0.39	0.92 ± 0.08	−0.94 ± 0.07	0.16 ± 0.05	3.54 ± 0.40	−0.43 ± 0.09	3.11 ± 0.42
E558A	1.08 ± 0.08	4.99 ± 0.11	−4.46 ± 0.11	0.11 ± 0.02	1.71 ± 0.09	−0.27 ± 0.07	1.44 ± 0.14
H428A	1.32 ± 0.21	−0.10 ± 0.31	0.29 ± 0.31	0.06 ± 0.01	1.57 ± 0.25	−0.18 ± 0.05	1.39 ± 0.28
K469A	1.10 ± 0.08	−3.41 ± 0.07	3.00 ± 0.07	0.09 ± 0.01	0.77 ± 0.08	−0.07 ± 0.02	0.69 ± 0.08
I331A	0.81 ± 0.08	−0.31 ± 0.12	0.31 ± 0.04	0.08 ± 0.05	0.88 ± 0.12	−0.38 ± 0.07	0.51 ± 0.17
L327A	0.41 ± 0.30	−0.02 ± 0.07	0.06 ± 0.07	0.04 ± 0.03	0.49 ± 0.31	−0.06 ± 0.05	0.43 ± 0.29
P451A	0.24 ± 0.15	0.03 ± 0.02	−0.01 ± 0.03	0.02 ± 0.02	0.28 ± 0.17	−0.04 ± 0.03	0.24 ± 0.15
F463A	0.22 ± 0.02	0.25 ± 0.03	−0.22 ± 0.04	0.00 ± 0.00	0.25 ± 0.04	−0.03 ± 0.01	0.23 ± 0.05
W427A	0.06 ± 0.00	−0.01 ± 0.02	0.12 ± 0.02	0.00 ± 0.00	0.17 ± 0.01	0.00 ± 0.00	0.17 ± 0.01
S470A	−0.60 ± 0.26	1.83 ± 0.29	−0.61 ± 0.11	0.00 ± 0.00	0.63 ± 0.10	−0.47 ± 0.08	0.16 ± 0.14
S328A	0.38 ± 0.15	0.65 ± 0.50	−0.73 ± 0.51	0.05 ± 0.04	0.35 ± 0.16	−0.26 ± 0.29	0.09 ± 0.17
Y559A	0.06 ± 0.01	−0.03 ± 0.01	0.06 ± 0.01	0.00 ± 0.00	0.09 ± 0.01	0.00 ± 0.00	0.09 ± 0.01
N557A	0.01 ± 0.00	0.04 ± 0.01	−0.04 ± 0.01	0.00 ± 0.00	0.01 ± 0.00	0.00 ± 0.00	0.01 ± 0.00
Q332A	0.69 ± 0.56	−0.39 ± 1.11	0.26 ± 1.15	0.07 ± 0.08	0.64 ± 0.57	−0.63 ± 0.73	0.01 ± 0.29
S430A	0.03 ± 0.01	−0.02 ± 0.07	0.01 ± 0.07	0.00 ± 0.00	0.02 ± 0.01	−0.02 ± 0.00	0.00 ± 0.01

*All energy values are given in kcal/mol. ΔΔE_vdw_, ΔΔE_ele_, ΔΔG_gb_, and ΔΔG_np_ represent the difference of van der waals interaction, electrostatic interaction, generalize-born and nonpolar solvation free energy between WT and the mutant*.

Decomposition of the binding free energy shows that PARP-1 and PARP-2 both have five hot-spot residues: Y907, Y889, Y896, H862, and E988 for PARP-1, and Y473, Y455, Y462, E558, and H428 for PARP-2.

**Figure 6:**
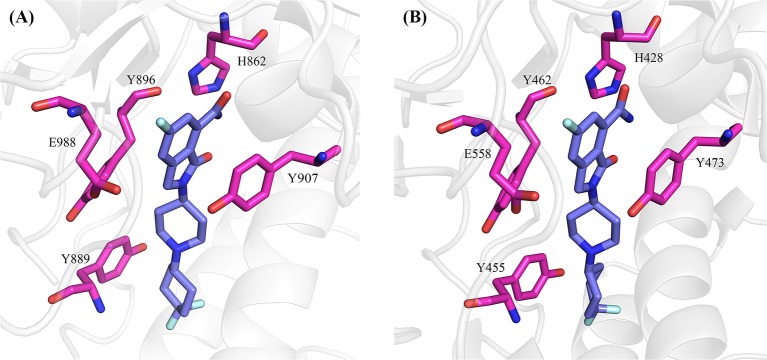
The hot-spot residues in PARP-1 (**A**, PDB ID: 5A00) and PARP-2 (**B**, PDB ID:4ZZY). The proteins are shown in cartoon, and the hot-spot residues and ligand are shown in sticks.

The positions of these residues in the two complexes are plotted side-by-side (Figure 6). The NMS-P118 occupies the nicotinamide-ribose sites90 (NI-sites) and displays a similar pose in the two complexes, and therefore forms similar interactions with PARP-1 and PARP-2. For example, it forms π-alkyl hydrophobic interaction with Y889 and Y896 in PARP-1 and Y455 and Y462 in PARP-2, and π-π stacking with Y907 in PARP-1 and Y473 in PARP-2.

**Figure 7:**
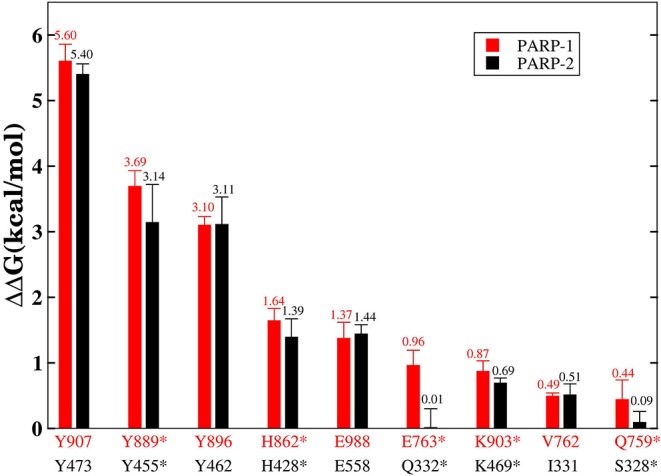
The residues that contributed the most to the binding free energy in PARP-1 and PARP-2. Red represents the residue in PARP-1 and black represents the residue in PARP-2. Stars indicate statistically significant difference (*t*-test, *p*-value < 0.01).

Although the two complexes have the same hot-spot residues, most of the residues in the PARP-1 have stronger binding free energy than the corresponding residues in PARP-2. To compare the difference between the corresponding residues in PARP-1 and PARP-2 clearly, the residues that contributed most to the binding free energy are shown in Figure 7.

**Figure 8:**
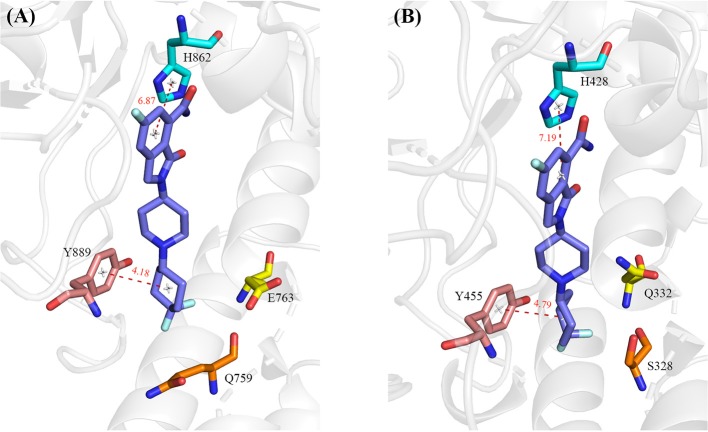
The hot-spot residues that are responsible for the different binding affinity in PARP-1 (**A**, PDB ID: 5A00) and PARP-2 (**B**, PDB ID: 4ZZY). The protein is shown in cartoon, and the residues and ligand are shown in sticks. The red dashed lines indicate distance between PARP1/2 residues and the ligand.

A number of hot-spot residues in PARP-1 such as Y889, H862, E763, and Q759 show stronger contributions to binding free energy compared to the corresponding residues in PAPR2, revealing the origin of the stronger binding of PARP-1 (Figure 8).

Y889 in PARP-1 contributes 0.55 kcal/mol more than the corresponding residue Y455 in PARP-2. We calculated the average distances between center of mass of benzene ring between Y889/Y445 and 4′4-difluorocyclohexyl in NMS-P118. The average distance of Y889 is 4.18 Å while the distance of Y445 in PARP-2 complex is 4.79 Å. The shorter distance in PARP-1 may account for the stronger van der Waals interaction of Y889 in PARP-1. H862 in PARP1 is 0.25 kcal/mol stronger than the corresponding residue H428 in PARP2. H862 is more strongly bound to the ligand because the distance with ligand is 6.87 Å, which is closer than the 7.19 Å distance of H428 in PARP2 complex. Moreover, a hydrogen bond exists between the ligand and backbone of H862 in PARP-1 and H428 in PARP-2. The occupancy of hydrogen in PARP-1 and PARP-2 is 54.31% and 44.14% in MD simulations, respectively, which can partly account for the stronger interaction in PARP-1 (Table 3). The binding free energy of E763 is 0.95 kcal/mol larger than that of Q332, which is likely due to the favorable electrostatic interactions of E763 and the positively charged 1,4-dimethylpiperidin-1-ium group in the ligand.

**Table 3:** Occupancy of hydrogen bonds between Q759/S328 and the ligand during MD simulation.

**System**	**Acceptor**	**Donor**	**Ave Distance (Å)**	**Ave Angle (°)**	**Occupancy (%)**
PARP-1	NMS-P118O28	H862CA-HA	3.50	144.92	54.31
	NMS-P118F27	Q759CB-HB3	3.08	126.79	77.93
	NMS-P118F26	Q759CA-HA	3.68	134.10	27.08
PARP-2	NMS-P118O28	H428CA-HA	3.55	147.82	44.14
	NMS-P118F27	S328CB-HB2	4.77	104.31	10.53
	NMS-P118F27	S328CA-HA	3.91	123.98	25.61

It is believed that the Q759 and V762 in PARP-1 and S328 and I331 in PAPP-2 are responsible for the selectivity of NMS-P118 in PARP-1 and PARP-2 (Papeo et al., 2015). However, our results show that there is no obvious difference between V762 and I331, and slightly stronger contribution from Q759 in PARP-1 than that of S328 in PARP-2. Therefore, the hydrogen bonds between ligand and Q759 in PARP-1 and S328 in PARP-2 are analyzed (Table 3). The occupancy of hydrogen bonds in PARP-1 are both larger than those of corresponding hydrogen bonds in PARP-2, which contribute to the larger binding free energy.

## Conclusion

The MM/GBSA_IE method that combines the MM/GBSA method and the IE method was used to obtain the quantitative binding free energy between NMS-P118 and PARP1/PARP2. Analysis of hot-spot residues explores the differences in the binding mechanism between NMS-P118 with PARP-1 and PARP-2.

PARP-1 has the same number of hot-spot residues with PARP-2, but the relative binding free energy of most hot-spot residues in PARP-1 is greater than that of the corresponding residues in PARP-2. Especially, Y889 in PARP-1 has 0.55 kcal/mol greater binding free energy than Y455 in PARP-2 because of the stronger π-alkyl interaction with 4′4-difluorocyclohexyl ring in ligand. Furthermore, H862 in PARP-1 is 0.25 kcal/mol stronger than H428 in PARP-2 because of the shorter distance with the ligand and more stable hydrogen bonds. Besides the stronger electrostatic interaction between E763 and positively charged ligand induces the 0.95 kcal/mol greater binding free energy than Q332 in PARP-2, which may also contribute to the selectivity. Moreover, the hydrogen bonds between Q759/S328 and 4′4-difluorocyclohexyl ring also cause the energy difference of 0.35 kcal/mol.

Selective PARP-1 inhibitors can not only be used as radiotherapy and chemotherapy sensitizers to enhance anti-tumor efficacy, but also would mitigate toxicities arising from cross-inhibition of PARP-2. The results of this study partly reveal the reasons for stronger binding of PARP-1 with NMS-P118, which may provide guidance for further improvement and design of potent selective PARP inhibitors.

## Data Availability Statement

The datasets generated for this study are available on request to the corresponding author.

## Author Contributions

RW performed main calculation study and wrote the draft paper. YC, ML, and JB helped coding the program and participated in discussion. YQ supervised and helped to write the paper. JZ designed and supervised the entire project.

### Conflict of Interest

The authors declare that the research was conducted in the absence of any commercial or financial relationships that could be construed as a potential conflict of interest.
